# HTLV-1/2 Infection in Blood Donors from a Non-Endemic Area (Catalonia, Spain) between 2008 and 2017: A 10-Year Experience

**DOI:** 10.3390/v14091975

**Published:** 2022-09-06

**Authors:** Maria Piron, Fernando Salvador, Estrella Caballero, Adrián Sánchez-Montalvá, Marta Bes, Natàlia Casamitjana, Lluís Puig, Israel Molina, Silvia Sauleda

**Affiliations:** 1 Banc de Sang i Teixits de Catalunya (Blood and Tissue Bank of Catalonia, BST), Transfusion Safety Laboratory, 08005 Barcelona, Spain; 2 Centro de Investigación Biomédica en Red de Enfermedades Hepáticas y Digestivas (CIBEREhd), Instituto de Salud Carlos III, 28029 Madrid, Spain; 3 Vall d’Hebron Research Institute (VHIR), Vall d’Hebron University Hospital, 08035 Barcelona, Spain; 4 International Health Unit Vall d’Hebron-Drassanes, Infectious Diseases Department, Vall d’Hebron University Hospital, PROSICS Barcelona, Universitat Autònoma de Barcelona, 08035 Barcelona, Spain; 5 Centro de Investigación Biomédica en Red de Enfermedades Infecciosas (CIBERINFEC), Instituto de Salud Carlos III, 28029 Madrid, Spain; 6 Laboratory of Microbiology, Vall d’Hebron University Hospital, 08035 Barcelona, Spain

**Keywords:** HTLV-1/2, screening, blood donors, leukoreduction, transfusion, Spain

## Abstract

Human T-cell lymphotropic virus type 1 and 2 (HTLV-1/2) screening is not mandatory in Spanish blood banks. In Catalonia, selective screening was introduced in 2008, followed by universal screening in 2011. We present herein a 10-year experience of HTLV testing in blood donors. HTLV-1/2 selective screening was performed using Ortho-Clinical Diagnostics HTLV-I/HTLV-II Ab-Capture ELISA between February 2008 and May 2009, then Abbott Prism HTLV-I/ HTLV-II assay until December 2010. Abbott Architect rHTLV-I/II assay was then used for HTLV-1/2 universal screening in pooled samples. INNO-LIA HTLV I/II Score (Fujirebio) and in-house HTLV-1/2 proviral DNA real-time PCR were used in reactive samples. Follow-up was offered to confirm HTLV-1/2 donors in Vall d’Hebron Hospital. Between 2008 and 2017, 51 blood donors were confirmed HTLV positive (46 HTLV-1, 4 HTLV-2 and 1 HTLV) out of 2,114,891 blood donations (1 in 41,468). Sixty-nine percent were female, median age was 40 years and most were born in Latin America (69%), followed by Europe (25%), Africa (4%) and Asia (2%). Screening of relatives and partners identified 12 additional HTLV-1 cases. Lookback studies did not show any HTLV-1/2 transmission. HTLV infections found in blood donors mirror epidemiological changes in the population of Spain. Consequently, HTLV should be considered a potential risk for recipients and calls for the design of optimal strategies to ensure transfusion safety.

## 1. Introduction

Human T-cell lymphotropic virus (HTLV) was the first oncogenic human retrovirus described [1]. HTLV is an intracellular retrovirus that infects T-cells and causes chronic infection in humans (for review [2,3]). HTLV type 1 (HTLV-1) and HTLV-2 are two closely related viruses responsible for the majority of HTLV infections globally. Two new types, HTLV-3 and 4, were later described in Africa, but cases are rare and little is known about these two pathogens.

HTLV-1 infection remains asymptomatic in most cases, but 5–10% of infected subjects eventually develop disease [4]. Infection with HTLV-1 is associated with two main entities: the aggressive and usually fatal adult T-cell leukemia/lymphoma (ATL), and the progressive neurodegenerative disease, HTLV-associated myelopathy/tropical spastic paraparesis (HAM/TSP). HTLV-2 pathogenicity has not been clearly established, although some studies associate HTLV-2 infection with the inflammatory disorder HAM/TSP [5,6]. There is neither a vaccine nor specific treatment for HTLV infection.

HTLV-1 affects five to ten million individuals worldwide, although this is probably underestimated as many countries do not report prevalence data [7]. The major HTLV-1/2 endemic regions, where seroprevalence ranges from 0.5–1% to almost 10%, are found in Japan, Africa, Central and South America, the Caribbean, Melanesia and Iran (for review [4,7]).

Transmission of HTLV requires cell-to-cell contact with infected lymphocytes. Three main routes of transmission have been established: (1) vertically from mother to child through prolonged breastfeeding, (2) by sexual contact, and more efficiently from male to female, and (3) by contact with substances of human origin containing infected lymphocytes (stem cells, tissue or organ transplantation). Transmission of HTLV-1/2, and especially HTLV-2, is described among injecting drug users [8,9,10,11,12]. In the absence of treatment, efforts must focus on HTLV-1/2 transmission prevention.

Depending on local epidemiology, various strategies are applied to reduce the risk of transfusion-transmitted HTLV-1/2. In some non-endemic countries, donor suitability is assessed through a pre-donation questionnaire, where individuals from HTLV-1/2 endemic countries are deferred from blood donation. Another strategy is serological HTLV-1/2 screening of blood donations, mandatory in many endemic and some non-endemic countries [13,14]. Finally, leukoreduction by filtration of blood components is applied in many blood banks, and allows removal of most of the HTLV-1/2 infective leukocytes. The latter strategy may be applied alone or in parallel with serological screening [14].

In Spain, leukoreduction by filtration of blood products was introduced in blood banks in 2002, but HTLV-1/2 screening is not mandatory. In Spanish blood banks, donor suitability regarding HTLV-1/2 infection risk is normally assessed through the pre-donation questionnaire; donors with known HTLV-1/2 infection or reporting residence in an HTLV-1/2 endemic country are deferred from donating blood.

Catalonia is an autonomous region situated in North-East Spain that, with 7.5 million inhabitants, represents 16% of Spanish population. All blood donors in Spain are volunteer and non-remunerated. The Blood and Tissue Bank of Catalonia (BST) is a government institution and the only blood bank in the region that provides all the hospitals with blood components. BST implemented HTLV-1/2 screening in 2008 as an additional safety measure to leukoreduction, initially in the same blood donors selectively tested for Trypanosoma cruzi (T. cruzi) infection (Chagas disease), mainly from Latin America, then in all blood donors through universal testing using pooled samples [15].

We present herein the results obtained with HTLV-1/2 testing in blood donors from a non-endemic area between 2008 and 2017.

## 2. Materials and Methods

### 2.1. Blood Donors and Study Design

Different HTLV-1/2 screening strategies and tests were applied in our blood bank between 2008 and 2017.

Between 2008 and 2010, in a first approach, selective individual screening for anti-HTLV-1/2 was performed in the same blood donors at risk for Chagas disease, endemic in continental Latin America. Individuals included belonged to one of the following risk groups: donors born or transfused in an endemic area, donors born of a mother native to a Chagas endemic area or residents in an endemic area for more than 1 month. All Latin America from Mexico to Chile was considered endemic.

After identifying the first HTLV-1/2 cases in the selected blood donors and in some of their sexual partners who did not originate from Latin America, the strategy evolved to universal testing. From 2011, HTLV-1/2 testing was performed in all blood donors, independently of their geographical origin, in a 48-sample minipool (MP48).

All blood donors answered and signed an ethics committee-approved questionnaire that included questions on risk factors related to HTLV-1/2 acquisition. Information on age, sex and place of birth was registered.

Repeatedly reactive donations in routine blood screening were tested with supplementary assays in the Transfusion Safety Laboratory, and the donors were contacted for retesting and counseling.

Confirmatory diagnosis, clinical follow-up and testing of family members and partners were offered to anti-HTLV confirmed positive blood donors in the International Health Unit of Vall d’Hebron University Hospital (Barcelona, Spain).

### 2.2. Screening and Supplementary Methods

#### 2.2.1. Anti-HTLV-1/2 Screening Methods

Three screening methods were successively used between 2008 and 2017. For the individual (ID) testing, serum samples were screened for the presence of HTLV-1/2 antibodies, first with the automated EC-approved assays HTLV-I/HTLV-II Ab-Capture ELISA Test System (Ortho-Clinical Diagnostics, Raritan, NJ; Triturus ELISA processor, Grifols Diagnostic, Barcelona, Spain), in place from February 2008 to May 2009, and then with the Abbott Prism HTLV-I/ HTLV-II chemiluminescent immunoassay (ChLIA, Abbott Laboratories, Diagnostics Division, Wiesbaden, Germany), in place from June 2009 to December 2010.

The minipool strategy (MP48) in all blood donations was validated between November 2009 and December 2010 using a third screening method, the Abbott Architect rHTLV-I/II chemiluminescent microparticles immunoassay (CMIA, Abbott, Diagnostics Division, Wiesbaden, Germany), in parallel with routine testing of individual selective blood donations using the Abbott Prism HTLV-I/ HTLV-II chemiluminescent immunoassay. An analytical sensitivity of 100% was previously observed after 48-fold dilution of positive samples from blood donors obtained in our blood center after HTLV-1/2 ID screening using a panel of 16 well-characterized HTLV-1 or HTLV-2 samples (Panel I B, data not shown), kindly provided by Vanderleia Barbaro and Simone Kashima (Hemocentro Ribeirao Preto, São Paolo, Brazil). For the validation, 100 µL EDTA plasma from each of 48 different blood donations were mixed to constitute a single 4.8 mL-sample using the automated Tecan liquid handling platform Freedom EVO Clinical (Tecan Group Ltd., Männedorf, Switzerland), which allowed full traceability of donations included in every MP48. Every reactive result observed in a pooled sample was resolved by individually testing the serum of each of the 48 donations using the Abbott Architect rHTLV-I/II assay.

During this validation period (November 2009–December 2010), blood donations considered at risk for Chagas disease were analyzed twice for anti-HTLV-1/2: in ID testing with the Abbott Prism HTLV-I/II assay, and in MP48 with the Abbott Architect HTLV-1/2 assay. The rest of the blood donations, not considered at risk for Chagas disease, were tested only once for anti-HTLV-1/2, in MP48 format.

HTLV-1/2 MP48 testing with Abbott Architect rHTLV-I/II was implemented after validation in all blood donations from 2011 to 2017.

#### 2.2.2. Supplementary Methods for HTLV-1/2 Initially Reactive Blood Donations

Samples that tested initially reactive for HTLV-1/2 antibodies in screening were retested twice and, if repeatedly reactive, were analyzed by HTLV-1/2 immunoblot (INNO-LIA HTLV I/II Score, Fujirebio, Gent, Belgium). The INNO-LIA HTLV I/II Score is a line immunoassay that confirms the presence of antibodies against HTLV-1/2 and, additionally, differentiates between HTLV-1 and HTLV-2. The results expected in this assay are: negative, indeterminate, positive for HTLV without typing (HTLV positive), positive for HTLV-1 or positive for HTLV-2. Furthermore, all initially positive samples were assessed for the presence of proviral HTLV-1 (TAX region) or HTLV-2 (LTR region) sequences using an in-house qualitative real-time polymerase chain reaction (PCR) for each HTLV region (modified from the protocol kindly provided by Vanderleia Barbaro and Simone Kashima, Hemocentro Ribeirao Preto, São Paolo, Brazil). Briefly, proviral DNA was obtained from 200 µL whole blood EDTA samples using the QIAamp DNA Mini Kit (Qiagen, Hilden, Germany) and eluted in 100 µL H_2_O. Five µL were used in each PCR reaction in a 25 µL final volume, using 1x TaqMan Universal PCR master Mix (Applied Biosystems, ThermoFisher Scientific, MA, USA), 0.1x RNase P Primer-Probe (VIC™ dye, Applied Biosystems, ThermoFisher Scientific, MA, USA), 600 nM each forward (FW) and reverse (RV) primer (HTLV-1 TAX FW: 5′-CCA TGC TTA TTA TCA GCC CAC TT; HTLV-1 TAX RV: 5′-CGT AGA CTG GGT ATC CGA AAA GA; HTLV-2 LTR FW: 5′-TCC GCG TTC TTG TCT CGT T; HTLV-2 LTR RV: 5′-GGC GTT GAG GTT TCG TTT TC) and 300 nM TaqMan FAM™ dye MGB probe (HTLV-1 TAX P: 5′-AGG GTT TGG ACA GAG TC; HTLV-2 LTR P: 5′-TTT CCT CTT CGC CGT CAC). Each PCR reaction was performed in a 7500 Real Time PCR System thermocycler (Applied Biosystems) and conditions were the same for both amplified regions: 2 min at 50 °C, 10 min at 95 °C, 40 cycles of 15 s at 95 °C and 1 min at 60 °C. A limit of detection for HTLV-1 proviral DNA of 3.8 HTLV-1 proviral copies/10,000 PBMC was obtained using a quantified HTLV-1 sample kindly provided by Dr. Ana Treviño and Dr. Vicente Soriano, Hospital Carlos III (Madrid, Spain). The results expected in this assay are: negative (HTLV proviral DNA not detected), positive for HTLV-1 or positive for HTLV-2. A frozen one-use aliquot of whole blood sample from donors previously confirmed as positive for HTLV-1 or HTLV-2 (immunoblot positive, PCR positive) was used as positive control for DNA extraction and HTLV specific detection in each PCR run.

The different combinations and interpretation of the results obtained using the supplementary assays are described in the following table (Table 1).

### 2.3. HTLV-1/2 Lookback

For regular blood donors who were identified in this study as HTLV-1/2 positive, a lookback procedure was initiated in all patients who had been transfused with blood products not tested for HTLV-1/2 or tested as nonreactive. Transfusion services in hospitals that were responsible for the blood component transfusion were contacted and asked to investigate the status of the recipients.

### 2.4. Statistical Analysis

For continuous variables, means with standard deviation (SD) were used. Anti-HTLV-1/2 prevalence were calculated with a 95% confidence interval (95% CI) using the Wilson score interval calculation method [16].

## 3. Results

### 3.1. HTLV 1/2 Selective and Individual Screening Strategy, 2008–2009

In a first approach, blood donors presenting a risk for Chagas disease and tested for anti-Trypanosoma cruzi were screened for HTLV-1/2. The most represented countries of origin of blood donors born in Chagas-endemic areas were Argentina (22%), Colombia (20%) and Ecuador (12%) followed by Brazil (10%), Peru and Uruguay (both 8%), Chile (7%), Venezuela (6%) and Bolivia (4%).

Between February 2008 and December 2009, 580,290 blood donations were collected in the blood center; of these, 26,604 were selectively and individually tested for HTLV-1/2 (4.6%, Table 2, section A). Twenty-seven were repeatedly reactive in the screening test (0.1%, 95% CI 0.07–0.15). Thirteen were confirmed HTLV-1 positive (5 from Peru, 2 from Bolivia, 2 from Chile, 2 from Ecuador and 2 from Colombia). During this first period, Peruvian blood donors accounted for 38% of HTLV-1 positive cases while representing only 8% of the Latin-American blood donors. The remaining 14 blood donations repeatedly reactive in the screening test were confirmed negative by immunoblot and proviral DNA PCR. The 2008–2009 prevalence observed in at-risk blood donors was therefore 0.05% (95% CI 0.03–0.08, or 1 in 2046, 95% CI 1 in 1196—1 in 3501) (Table 2, section B).

### 3.2. Validation of HTLV-1/2 Universal Minipool (MP48) Testing Strategy, 2010

During 2010, a pooled sample testing strategy was evaluated, in parallel to the selective-ID screening already in place, to universally test all blood donations independently of the donor’s geographical origin.

From January to December 2010, 290,717 blood donations were analyzed in 6102 MP48 samples (Table 2, section A). Seven MP48 out of 6102 (0.11%) were reactive in the HTLV-1/2 test. One reactive blood donation was identified in each initially reactive MP48. Two of the seven initially reactive blood donations obtained from two Spanish blood donors were indeterminate in the immunoblot study, with undetectable proviral DNA. The other five were confirmed HTLV positive: two confirmed HTLV-1 positive, first-time blood donors, were from Peru (female, 56 years at donation) and Ecuador (male, 23 years). The other three positive donors were from Spain, all of them female repeat blood donors (55 years: HTLV-1; 66 years: HTLV-1 and 51 years: HTLV). The prevalence observed in all blood donors in 2010 MP48 testing was therefore 0.002% (95% CI 0.001–0.004, or 1 in 58,143, 95% CI 1 in 24,836—1 in 136,122) (Table 2, section B).

During the same period, parallel HTLV-1/2 selective ID screening of at-risk blood donors was performed in 25,689 blood donations using the Abbott Prism HTLV-1/2 assay (Table 2, section A). Twenty blood donations were repeatedly reactive (0.08%) in ID screening. Of these, two, obtained from blood donors from Peru and Ecuador, were confirmed HTLV-1 positive and also identified through MP48 testing described in the previous paragraph (Table 2, section B). Fourteen were negative in immunoblot and proviral DNA PCR, and four were indeterminate in the immunoblot, with undetectable proviral DNA.

The three Spanish blood donors could only be identified through the universal MP48 testing since they did not present any risk for T. cruzi infection and had not been included in the parallel selective-ID testing.

From these results, we were able to show that, compared with selective-ID testing, universal MP48 testing presents a higher yield associated with higher coverage (five positive donors identified versus two in selective-ID screening) and higher specificity (zero false positive out of seven initially reactive in MP48 versus fourteen false positive out of twenty initially reactive in ID testing) (Table 2, section B). In our low HTLV seroprevalence context, only 7 MP48 out of 6,102 had to be opened during the parallel evaluation, which allowed 290,717 blood donations to be analyzed using fewer than 6500 tests, while ID-selective strategy required 25,689 tests and only allowed a part of the blood donations to be analyzed. Furthermore, the high throughput of the Architect analyzer enables initially reactive MP48 samples to be rapidly resolved and to identify the positive blood donation(s) in the MP48.

### 3.3. HTLV-1/2 Universal MP48 Testing Results, 2011–2017

Once validated, universal MP48 HTLV-1/2 screening was implemented in 2011 for all blood donors in Catalonia, independently of their geographical origin. Between 2011 and 2017, this strategy led to the identification of 29 HTLV-1 and 4 HTLV-2 positive blood donors. The prevalence observed in all blood donors between 2011 and 2017 using MP48 testing was therefore 0.0018% (95% CI 0.0013–0.0025, or 1 in 55,278, 95% CI 1 in 39,362—1 in 77,628) (Table 2, section B).

### 3.4. Summary of HTLV-1/2 Positive Blood Donors Identified between 2008 and 2017

From February 2008 to December 2017, 51 blood donors were confirmed positive for HTLV-1/2 out of 2,114,891 blood donations tested for anti-HTLV-1/2 (1 in 41,468; 95% CI 1 in 31,543—1 in 54,518). Forty-six were positive for HTLV-1, four were positive for HTLV-2 and one blood donor was confirmed as HTLV positive without type differentiation (Table 3). Mean age at donation was 40 years (SD 13 years), ranging from 18 years to 66 years, and 69% were female.

During this period, 11 blood donors were classified as “Indeterminate”, with an initial reactive screening result and an indeterminate immunoblot result (all with HTLV PCR negative result). Follow-up with new blood sample extraction was performed in nine of them. Follow-up was performed by the blood bank in the months or even 1 year following the initial reactive blood donation and, in some cases, follow-up was additionally performed by the International Health Unit of Vall d’Hebron University Hospital. No evolution of HTLV status could be observed in any case that could evidence a seroconversion to HTLV.

As for the origin of positive blood donors, Latin American donors accounted for 67% of positive donors, while 25.5% of HTLV-1/2 positive donors were born in Europe. Interestingly, three out of four HTLV-2 cases as well as the non-typed HTLV donor were born in Spain (Table 2).

HTLV proviral DNA was detected in 46 of the 51 HTLV-1/2 blood donations (90% positive PCR). In one of these 46 PCR positive cases, immunoblot was indeterminate but proviral DNA PCR identified the virus as HTLV-2. Donations with undetectable HTLV DNA (5 out of 51) were identified by immunoblot as HTLV-1 in four cases or confirmed as HTLV without type differentiation in one case (Table 4).

Thirty-nine out of the 51 HTLV-1/2 positive blood donors were first-time blood donors. Conversely, 12 blood donors had given blood before the HTLV-1/2 positive screening result. Furthermore, two of them seroconverted to HTLV-1 during the study period. One of these two donors had tested nonreactive for HTLV-1/2 on two occasions using ID-selective screening, and became HTLV-1 positive 3 years after the last nonreactive result when tested using MP48 screening. The second case of seroconversion was detected 6 years after the last blood donation with a nonreactive result.

### 3.5. Lookback Investigations of Recipients Transfused with Blood Products from HTLV-1/2 Positive Donors

Ten donors out of fifty-one were regular blood donors when HTLV screening was implemented and previous donations had not been tested for HTLV-1/2. As HTLV-1/2 infection could have been acquired previously, all of these blood donations could therefore potentially have transmitted the virus. Additionally, two of the fifty-one positive blood donors seroconverted to HTLV-1 during this study and, although tested HTLV-1/2 nonreactive at the time of previous blood donation, the recipients were investigated. In total, 38 blood donations had been obtained from these 12 HTLV-positive repeat blood donors between 1991 and 2011. Fifty-two components associated with these 38 donations were issued for transfusion (34 red blood cell concentrates [RBC], 16 platelet concentrates [PLT] and 2 plasma units [PL] treated for pathogen reduction with methylene blue) and the lookback procedures were initiated (Table 5). Information was received from hospitals in 31 cases. One RBC and two PLT units expired in hospital before use, while eight PLT and fourteen RBC recipients had died when lookback was performed. Three RBC, two PLT and one PL recipients were able to be contacted and tested nonreactive for HTLV-1/2 in the follow-up sample; all had received leukoreduced components. Finally, regarding the other 21 products, we were unable to obtain any information from the hospitals (16 RBC, 4 PLT and 1 PL recipients). Fifteen out of these twenty-one components were issued after 2002 and had thus been leukoreduced, while six of them had been issued before 2002 and leukoreduction was not performed (4 RBC and 2 PLT).

### 3.6. Clinical Diagnosis and Follow-Up of HTLV Positive Blood Donors and Their Relatives

Of the 51 HTLV-1/2 positive blood donors, 7 could not be traced back and 5 received information about their HTLV-1/2 infection from the blood bank, but no clinical follow-up data was available, as donors declined the appointment arranged in the International Health Unit of Vall d’Hebron University Hospital. Information was available for 39 out of 51 HTLV-1/2 positive blood donors. Blood donors were tested again for HTLV antibodies according to the hospital microbiology laboratory procedure (EIA Murex HTLV I+II, Diasorin, Saluggia, Italy) and confirmed by line immunoassay (INNO-LIA HTLV I/II Score, Fujirebio, Gent, Belgium). HTLV infection was confirmed in all 39 patients (data not shown).

None of the donors stated that they were a current or former intravenous drug user and five (12.5%), all from HTLV-1/2 high prevalence countries, declared that they had previously received a blood transfusion in their country of origin, information not disclosed in the blood bank questionnaire. Overall, 54 relatives and sexual partners were tested for HTLV infection, 12 of whom were positive: 7 sexual partners (from 7 different donors), 1 sibling (sister), 1 daughter, 1 father, and 2 mothers. In 6 HTLV-1 positive couples, both partners were born in an endemic country (Peru, Ecuador or Colombia). In one couple, the donor was born in Chile while her partner was born in Spain (Table 6).

The HTLV-1 seroconversion observed in a Colombian blood donor between 2010 (last HTLV-1/2 nonreactive blood donation) and 2013 (first HTLV-1 positive blood donation) was probably associated with the start of a new relationship soon after the last negative blood donation with an HTLV-1 positive Colombian partner. As for the other seroconversion case in an Ecuadorian donor between 2011 and 2017, the only available information was that the sexual partner was also identified HTLV-1 positive during the hospital follow-up. All the blood donors and relatives were completely asymptomatic at the time of the clinical evaluation.

## 4. Discussion

This study performed in blood donors and their relatives identified 63 HTLV-1/2 asymptomatic patients in Catalonia in 10 years. HTLV-1/2 infection was probably acquired by vertical or sexual transmission routes in most of the cases, while we cannot rule out transmission by transfusion in the country of origin in five cases. The five blood donors who reported a previous blood transfusion during the clinical follow-up would not have been eligible for blood donation if they had reported this transfusion history in the pre-donation blood bank questionnaire, as a transfusion performed in any of their birth countries (Chile, Colombia, Ecuador, Romania and Iran) is a reason for deferral in our blood center. In some cases, sexual transmission was suspected but unfortunately the sexual partner could not be tested: this applied to two HTLV-1 positive donors born in a non-endemic area (Spain, Germany) who reported having had a sexual partner born in an endemic area (the Caribbean and Sub-Saharan Africa, respectively). Finally, three out of the four HTLV-2 blood donors were Spanish. Most cases of HTLV-2 infection are identified in Spain among intravenous drug users, and most of the cases are actually inmates, coinfected with HIV-1, identified in Spanish prisons [17,18]. Former or current intravenous drug users are not eligible for blood donation, but none of the four HTLV-2 blood donors reported this risk behavior in the blood donation questionnaire. Two of them agreed to clinical follow-up and were asked again about this risk factor, but definitively denied any intravenous drug use.

The lookback study was performed in 12 out of 51 (23.5%) positive blood donors with previous history of blood donation and did not demonstrate any HTLV-1/2 transmission by blood transfusion. Information was received on 31 out of 52 components (60%). This rather low lookback efficiency may be explained by the difficulty in obtaining information from hospitals many years after transfusion. The recipient’s status was not reported for 21 components, 6 of which had been obtained before 2002 and were transfused between 1991 and 1997 (13 to 19 years before lookback was performed). Only a small number of recipients could be tested for HTLV, as very few of them were still alive at the time the lookback was performed. All the living recipients had received leukoreduced components and tested negative for HTLV. The high efficacy of leukoreduction in reducing the risk of HTLV-1/2 transmission was previously shown in a large study in the United Kingdom [19], and, although the leukocyte acceptance limit of a maximum 10^6^ cells/bag after leukoreduction may be sufficient to transmit the virus from a donor with a very high proviral load, the likelihood of transmitting HTLV-1 by transfusion of leukoreduced components remains very low [20,21,22].

In Spain, new cases of HTLV-1/2 infection are annually reported by the Spanish HIV-2/HTLV Study Group. Between 1985 and 2007, fewer than five cases per year were identified in the entire country (105 cases in 22 years [17]), most of which were diagnosed in hospitals after the onset of symptoms. HTLV-1/2 prevalence was shown to be quite low in Spain, although some studies performed in high-risk populations indicated a higher prevalence in individuals from endemic regions [18,23].

This low prevalence appears to contradict the justification for screening for HTLV-1/2 infection in blood banks, especially because universal leukoreduction of all blood components was introduced in Spain in 2002. Leukoreduction reduces the HTLV-1/2 proviral load and HTLV-1/2 transmission risk by retaining infected lymphocytes. Moreover, regional blood banks comply with the Spanish Ministry of Health’s recommendation and defer potential blood donors proceeding from highly endemic countries.

Massive immigration from Latin America to Spain began in the 2000s, and prompted Spanish blood banks to take measures to manage the resulting demographic and epidemiological changes. In 2005, a Spanish Royal Decree for blood donation made T. cruzi infection screening mandatory for all individuals born in a Chagas disease-endemic area or whose mother was born in an endemic area [15]. As most of these areas also had a moderate or high prevalence of HTLV-1/2 infection, our blood bank decided in 2008 to perform HTLV-1/2 screening in the same blood donors, as an additional safety measure. The identification of the first HTLV-1 cases, and of an HTLV-1 positive Spanish sexual partner during follow-up, led us to consider HTLV-1/2 screening in all blood donors using pool testing. HTLV-1/2 testing using sample pools had previously been evaluated and implemented in United Kingdom blood banks [24,25,26]. This strategy was optimal to reduce costs in reagents, technician time and indirectly in confirmatory tests, with the method being more specific as borderline reactive results were not observed.

In non-endemic countries, blood bank HTLV-1/2 screening, as an additional safety measure to leukoreduction, is regularly challenged, as some consider it redundant and expensive [22]. In our blood bank, the use of sample pools substantially reduces the number of false-positive results and the cost of testing, making it affordable and highly beneficial for transfusion safety. As an alternative strategy, HTLV selective-ID screening in first-time blood donors may be considered, but this would miss incident seroconversion cases, which we observed in our cohort.

Our results highlight an epidemiological change in Spain in terms of HTLV-1/2 prevalence that should be taken into consideration in blood banks, although the transmission risk through transfusion remains relatively low after leukoreduction of blood components. The combination of leukoreduction and pool testing remains perhaps the most optimal option regarding HTLV transfusion safety.

## Figures and Tables

**Table 1:** Interpretation of all possible supplementary assays results combinations used to establish HTLV infection status in screening reactive donors.

		PCR (HTLV-1 or-2 Proviral DNA) Results		
		HTLV-1(+)	HTLV-2(+)	Negative
**Immunoblot (anti-HTLV-1/2) results**	HTLV-1(+)	HTLV-1(+)	-	HTLV-1(+)
	HTLV-2(+)	-	HTLV-2(+)	HTLV-2(+)
	HTLV(+)	HTLV-1(+)	HTLV-2(+)	HTLV(+)
	INDETERMINATE	HTLV-1(+)	HTLV-2(+)	INDETERMINATE
	NEGATIVE	HTLV-1(+)	HTLV-2(+)	NEGATIVE

**Table 2:** HTLV-1/2 testing methods (A) and results (B) by period, February 2008–December 2017.

**A**	**Period**	**Feb.2008–Dec.2009**	**Jan.–Dec.2010** **(Parallel Screening)**		**Jan.2011–Dec.2017**
	Screening strategy	ID-selective	ID-selective	MP48-universal	MP48-universal
	Assay	Ortho-Clinical HTLV-I/II Ab Capture ELISA Test System	Abbott Prism	Abbott Architect	Abbott Architect
	N total blood donations	580,290	290,717	290,717	1,824,174
	N HTLV-1/2 tested blood donations	26,604	25,689	290,717	1,824,174
**B**	**Period**	**Feb.2008–Dec.2009**	**Jan.–Dec.2010 (Parallel** **screening)**		**Jan.2011–Dec.2017**
	N HTLV-1/2 RR donations	27	20	7	42
	% HTLV-1/2 RR blood donations	0.10	0.08	0.002	0.002
	HTLV-1/2 confirmed negative (immunoblot and PCR negative)	14	14 *	0	4
	HTLV-1/2 confirmed indeterminate (PCR negative)	0	4 *	2 **	5
	HTLV confirmed positive (PCR negative)	0	0	1 **	0
	HTLV-1 confirmed positive (immunoblot and/or PCR positive)	13	2	4 ***	29
	HTLV-2 confirmed positive (immunoblot and/or PCR positive)	0	0	0	4
	**Total HTLV-1/2 confirmed positive per period**	**13**	**5**		**33**
	**HTLV-1/2 prevalence in tested donations per** **period (95%CI)**	**1/2046 (1196–3501)**	**1/58,143 (24,836–136,122)**		**1/55,278** **(39,362–77,628)**
	N HTLV-1/2 confirmed positive 2009–2017	51			
	N HTLV-1/2 tested blood donations 2009–2017	2,114,891			
	**HTLV-1/2 prevalence in tested donations (95%CI)**	**1/41,468 (31,543–54,518)**			

A: HTLV-1/2 testing strategies used during the three study periods and number of tested blood donations. B: HTLV-1/2 repeatedly reactive (RR) blood donations and confirmatory results. HTLV-1/2 prevalence in tested donations, for each period and overall. Donors were classified in 5 categories regarding HTLV infection status: negative, indeterminate, HTLV positive, HTLV-1 positive or HTLV-2 positive following immunoblot and PCR results combinations (see Table 1). N: number; ID: individual testing; MP48: 48-sample minipool testing; selective: testing in at-risk donors; universal: testing in all donors; *: all of them were negative in MP48-universal parallel screening; **: samples not included initially in the ID-selective parallel screening, ***: two of them were also RR in ID-selective parallel screening.

**Table 3:** HTLV-1/2 positive blood donors according to their birth country (2008–2017).

Country of Birth	Number HTLV-1/2 Positive		
	HTLV-1	HTLV-2	HTLV
**Latin America**			
Bolivia	4 *	1	
Chile	4		
Colombia	5 **		
Dominican Republic	1		
Ecuador	6 **		
Honduras	1		
Peru	12		
Venezuela	1		
**Europe**			
France	1		
Germany	1		
Romania	3		
Spain	4	3	1
**Africa**			
Morocco	1		
Senegal	1		
**Asia**			
Iran	1		
**Total HTLV by type**	**46**	**4**	**1**

*: one *Trypanosoma cruzi* coinfection; **: one HTLV-1 seroconversion.

**Table 4:** HTLV-1/2 supplementary tests results of the 51 positive blood donors.

		PCR (HTLV-1 or-2 Proviral DNA)		
		HTLV-1(+)	HTLV-2(+)	NEG
**Immunoblot (anti-HTLV-1/2)**	HTLV-1(+)	41	0	4
	HTLV-2(+)	0	3	0
	HTLV(+)	1	0	1
	INDETERMINATE	0	1	0

**Table 5:** Lookback of the 52 blood components previously obtained from undiagnosed HTLV blood donors.

		RBC	PLT	PL
N not informed by Hospital Transfusion Service (HTS) (*)		**16 (4)**	**4(2)**	**1(0)**
**N informed by HTS: expired before use**		**1**	**2**	**0**
**N informed by HTS: component transfused**		**17**	**10**	**1**
Recipient status:	Deceased (*)	14(2)	8(0)	0
	HTLV negative (*)	3(0)	2(0)	1(0)
	HTLV positive	0	0	0
**N total issued components**		**34**	**16**	**2**

RBC: red blood cells; PLT: platelet concentrate; PL: plasma; N: Number; *: number of non-leukoreduced components in parentheses.

**Table 6:** HTLV-1(+) relatives or sexual partners of HTLV-1(+) blood donors.

HTLV-1(+) Blood Donor ID	Sex (Female/Male)	Age (Years)	Birth Country	HTLV-1(+) Relative or Sexual Partner (Birth Country)	Other Relative Tested HTLV Negative
2	F	55	CHILE	Sexual partner (SPAIN)	-
3	M	49	PERU	Sexual partner (PERU)	2 children
5	F	51	PERU	Sexual partner (PERU)	2 children, 5 siblings
6	F	18	ECUADOR	Mother and father (ECUADOR)	1 sibling
9	M	52	PERU	Sexual partner (PERU)	-
33 *	F	41	COLOMBIA	Sexual partner (COLOMBIA)	-
38	F	28	BOLIVIA	Daughter (SPAIN)	-
39	M	20	PERU	Mother and sister (PERU)	-
44	M	57	PERU	Sexual partner (PERU)	2 children
51 *	F	34	ECUADOR	Sexual partner (ECUADOR)	-

*: Donors who seroconverted to HTLV-1 during this study.

## Data Availability

Data from the validation of anti-HTLV minipool testing, immunoblot results and PCR are available upon request.
